# From Hydra Regeneration to Human Brain Structural Plasticity: A Long Trip through Narrowing Roads

**DOI:** 10.1100/tsw.2011.113

**Published:** 2011-06-09

**Authors:** Luca Bonfanti

**Affiliations:** Department of Veterinary Morphophysiology and Neuroscience Institute Cavalieri Ottolenghi (NICO), University of Turin, Turin, Italy

**Keywords:** stem cells, tissue regeneration, tissue repair, cell proliferation, cell renewal, adult neurogenesis, glial cells, neurons, immune system, neurodegenerative diseases

## Abstract

Regeneration is a strategy to maintain form and function throughout life. Studies carried out on animal models throughout the phylogenetic tree have flourished in the last decades in search of mechanisms underlying the regenerative processes. The development of such studies is strictly linked with stem cell research and both are viewed as one of the most promising outcomes for regenerative medicine; yet, regeneration, stem cells, and tissue repair do not seem to follow a logical path through the different animal species and tissues. As a result, some mammalian organs, e.g., kidney and brain, have lost most of their regenerative capacity. The human nervous system, although harboring neural stem cells, is placed at the extreme of “perennial” tissues. In addition, it is affected by neurodegenerative diseases, whose heavy burden is heightened by enhanced life spans. This review, starting from the basic principles of tissue regeneration viewed in a comparative context, tries to answer this question: To which extent can regenerative medicine be figured out in a mammalian brain equipped with many anatomical/evolutionary constraints?

### INTRODUCTION

The word "regeneration", particularly recurrent in recent biomedical literature, is often associated with the words "stem cells". Both concepts are fundaments of the so-called regenerative medicine, elected as the future of modern medicine. The principle at the basis of regenerative medicine is a very simple and strong one: to help tissues and organs to repair/heal by themselves, using their endogenous (sometimes quiescent) regenerative potentialities[1]. In many cases, animal tissues and organs already do this constitutively; for instance, labile tissues, such as some epithelia, undergo cell renewal physiologically and do regenerate after moderate lesions (e.g., skin[2]). Nevertheless, in complex organisms, not all tissues are endowed with the same capability for cell renewal and self-repair. This concept can be easily seized by looking at two simple facts: the different regenerative capacities of animals through the phylogenetic tree, and the different capabilities of cell renewal in different tissues and organs in individuals of the same species. Thus, in most organs of mammals, including humans (complex metazoans), the regenerative potential of cells and tissues is not enough to grant healing, so that it is a common statement that we have lost, to a great extent, the capacity for regeneration[3,4]. At the extreme of this loss is the most complex and intriguing system of the body, made up of substantially “perennial” tissue: the nervous system[5,6,7].

In the last few years, the field of “comparative regeneration” has flourished in parallel with an exponential increase in stem cell studies[8]. For a long time, many simple organisms have been viewed as (and indeed are) excellent models for investigating regeneration as a biological process[9,10] (reviewed in [11]). In parallel, the discovery of stem cell compartments in many mammalian tissues and organs has sometimes produced illusory hopes that similar potentialities might be easily manifested in complex organisms as humans are, by “awakening” the same regenerative processes. For instance, in neurobiology, breakthroughs revealing the occurrence of neural stem cells in the adult mammalian brain[12] and the consequent persistence of neurogenesis[13] have demolished the dogma of the nervous system as a nonrenewable tissue. Such revolutionary advancements have raised the attractive hypothesis that regenerative medicine could apply to traumatic, vascular, and neurodegenerative pathologies of the nervous system[14,15,16]; yet no substantial, efficacious, therapeutic approaches of cell replacement are at present available for most of those pathologies.

In this review, some aspects of regeneration/repair will be analyzed at different levels, e.g., under their evolutionary (in different animal species) and anatomical (in different tissues) profiles, trying to summarize why and how such capacities have progressively decreased in parallel with increased complexity in metazoans, such as mammals. At the end of this trip through regeneration in the animal world, we will try to define which potentialities (and limits) actually exist within our brain.

### REGENERATION IN THE ANIMAL WORLD

#### What is Regeneration?

Regeneration is the best strategy to maintain form and function throughout life. Histologically, it is a process that restores the interrupted continuity of a missing organ mass, yielding new fully functional tissue (perfect regeneration). However, many complex tissues undergo repair instead of regeneration; namely, they adapt to loss of normal tissue through its restoration by scarring (improperly referred to as imperfect regeneration). Thus, regeneration restores the normal structure and function of the organ, whereas repair does not[1,17,18].

When generally speaking of regeneration, we refer to heterogeneous biological events in which several different processes can be involved depending on the species, organ, tissue, and age. For instance, reconstitution of an organ through a blastema of undifferentiated cells (e.g., limb regeneration in amphibians) is highly different from local repair by restoration of one cell type (e.g., skeletal muscle; see below). It is generally admitted that spontaneous regeneration is linked to whatever potentially embryonic does remain within the adult body, somehow recapitulating developmental programs and showing complex interactions with the tissue architectures existing in mature organisms[18,19,20,21]. For this reason, regeneration has also been defined as an example of postembryonic morphogenesis[22] that appears to be strictly related to phylogenesis[7,22]. As a consequence, the road traveled by regenerative processes through evolution is not a linear one, exiting in complete loss of such capacities in some cases, or in a complex overlapping with tissue repair processes in others.

In order to gain insight into the intermix of the above-mentioned aspects, during the last few years, a phylogenetic approach to the issue of regeneration has rapidly expanded, starting from the long-lasting knowledge that many invertebrates and some vertebrates can regenerate whole body parts after their loss[9,10]. In spite of substantial advances in the understanding of mechanisms and modulators of regeneration (reviewed in [1,11,22]), many issues remain unresolved. In the following paragraphs, some aspects of regeneration in different animal species and tissues will be synthesized, starting from the most simple question: Why do some animals regenerate, whereas others do not?

### Regeneration in Different Phyla

An almost unlimited regeneration capacity is restricted to some living organisms. Plants and some phylogenetic members of the animal kingdom (e.g., cnidarians, echinoderms, several types of worms) share remarkable features that are not found in more complex animals[11,23]. Many invertebrate phyla are capable of regenerating whole body parts on both the dorsoventral and mediolateral planes (Fig. 1). Among cnidarians, the freshwater polyp hydra is one of the most well-understood models of regeneration among invertebrates[24]. Among worms, planarians can regenerate whole animals from tiny fragments from almost any part of their bodies after amputation[25]. Also, many members in the phylum of echinoderms (e.g., crinoids, including feather stars and sea lilies) possess high potentials, being able to regenerate most of their organs[26,27]. Such capabilities are lost in vertebrates, although large body parts can still be rebuilt in some species; the most striking case being that of amputated limbs in urodele amphibians[28,29] and tail regeneration in lizards[30]. Other nonmammalian vertebrates, e.g., teleost fish, have a great capacity to regenerate a variety of tissues and organs, including the heart, muscles, fins, and spinal cord[31,32,33,34]. Unlike amphibians and fish, mammals have a far more reduced capacity for regeneration[3,4]. This discrepancy seems not to be just a matter of complexity, since relatively simple organisms, such as *Drosophila* or *Caenorhabditis elegans*, have limited regenerative capacities[35], and many structures/organs of amphibians and fish are strikingly complex.

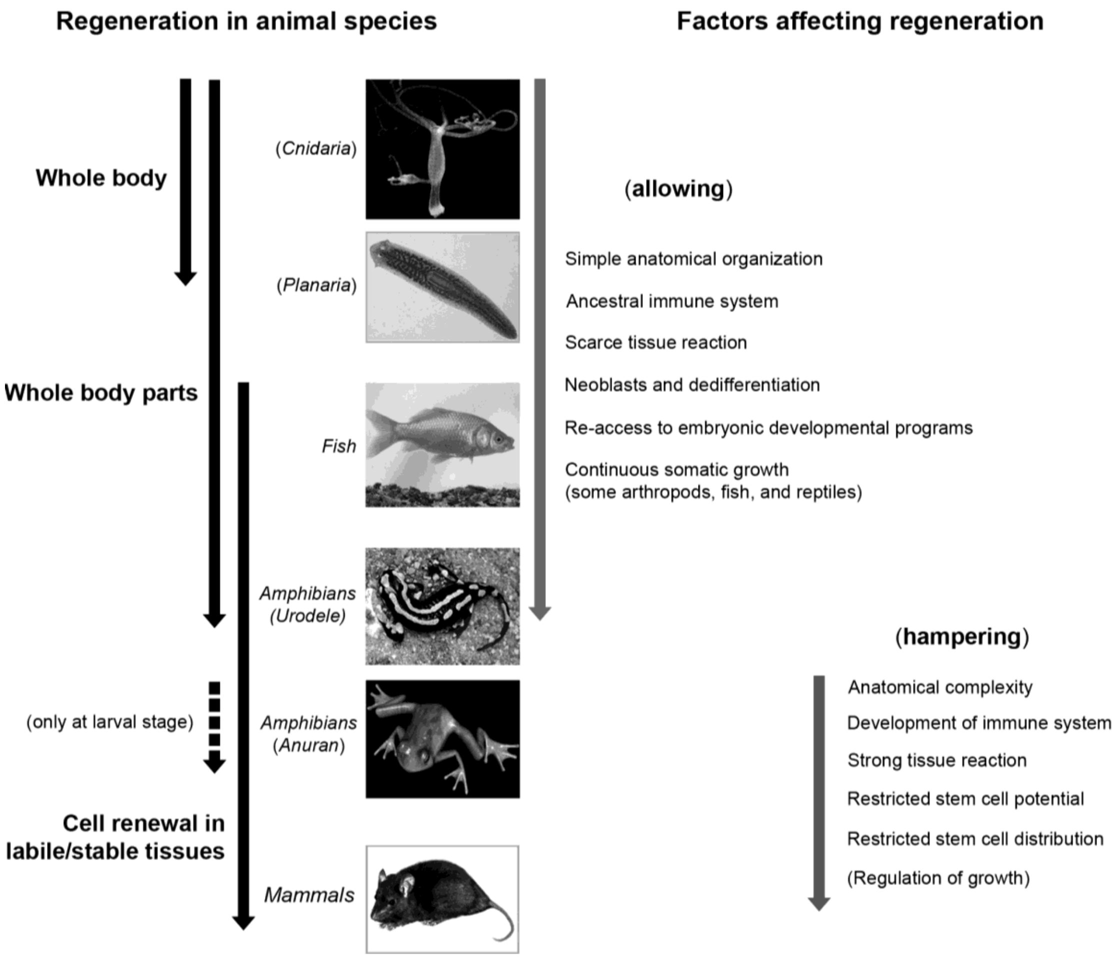

**FIGURE 1.** Schematic representation of the progressive restriction of regenerative capacity in different animal species, put in relation with several variables.

Classic studies have classified regeneration as "epimorphic", in which a blastema of undifferentiated cells is formed, and "morphallactic", in which direct rearrangement of pre-existing cells is observed. Amphibian limb regeneration is a typical example of epimorphic regeneration[29], whereas morphallaxis occurs in hydra[24]. However, planaria regeneration appears to be a mix of epimorphic and morphallactic processes, thus suggesting that old definitions should be reconsidered after studying the process at the cellular level. More accurate cellular and molecular studies in several regeneration models, including planaria[36] and amphibians[37], revealed the occurrence of "intercalary" regeneration, in which a blastema is first formed to act as a signaling center to reorganize body regionality, then stem cells start to differentiate in order to provide the appropriate cell types[38].

A growing number of comparative studies has recently updated our knowledge about different regeneration factors at the genetic, molecular, and cellular level across the animal kingdom (references in [8,39,40]). The involvement of two subsequent steps is a common aspect of regeneration. The first step is the recognition of tissue loss or injury, and this depends on different stimuli (e.g., bioelectrical signals, thrombin activation). The second step is the activation of cells, usually leading to formation of a wound epithelium. The origin of activated cells is still debated; both reserves of undifferentiated cells and differentiated cells endowed with plasticity can be involved[22,41]. Unfortunately, dedifferentiation is one of the issues that has not yet been resolved in stem cell research, being a feature that also highly varies in the phylogenetic tree[42]. Skeletal muscle could be a prototypical example; regeneration in the whole amphibian limb reconstitution occurs through dedifferentiation, whereas in mammals, a moderate muscle regeneration starts from satellite cells[43]. In the case of the myocardium, in humans, heart damage is followed by fibrosis and scarring, whereas heart regeneration with replacement of lost contractile tissue does occur in zebrafish and newt[41,44]. In these species, the new myocardium seems to arise from cardiomyocytes that re-enter the cell cycle through dedifferentiation rather than from undifferentiated stem/progenitor cells[45,46].

On the basis of concepts expressed above, the rationale for understanding why regenerative capacities are either maintained or lost seems to reside in the lifelong availability of stem/progenitor cells. Nevertheless, the stem cells are not always so plastic in the increasing complexity of adult vertebrate tissues, and the studies on the link between stem cells and regeneration across different phyla are still fragmentary.

### Stem Cells and Regeneration

It is generally recognized that the regenerative ability in invertebrates and some vertebrates is basically granted by a population of pluripotent stem cells, called "neoblasts", which can be found throughout the body[36,38,47]. On the other hand, in most mammalian tissues, the cell sources of regeneration are represented by stem/progenitor cells associated with the tissue itself[3].

Stem cells have been described for animals, fungi, and plants, and are probably a basic feature of all multicellular organisms. All stem cell systems share the universal property of continuously reproducing themselves and generate progeny of differentiated cells, a fact that also is central for stem cell function at the different levels (whole body, organs, tissues) of the various multicellular organisms. In simpler organisms, such immortality has been ascribed to the asexual mode of reproduction that requires cells with an unlimited self-renewal capacity[24]. It has also been proposed that the basic properties of animal stem cells are linked to this archaic mode of reproduction; yet, in this case, adapted to new, increasingly complex environments. For instance, in some echinoderms (asteroids), detached body fragments can regenerate to produce new complete adults in a sort of cloning strategy that is a mix of regeneration and reproduction[26].

Thus, a question can arise concerning how stem cells, tissue architectural complexity, and regeneration can be related, and if a shared logic can be found among these processes under a comparative profile. The activity of stem cells in adult organs can be considered as a theme with many variations, in which stem cells adapt to different maturing tissues by establishing their niches in a process depending on several variables[48]. As stated in the above sections, intact biological tissues show heterogeneous capacities for spontaneous cell renewal, which is very high in labile tissues (such as skin, blood) and very low or absent in perennial tissues (such as the brain and skeletal muscle of mammals[49]). These distinctions become less sharp if considering simpler metazoan organisms[8,25,29,43,50]. Since in most tissues/organisms the endogenous cell renewal activity seems to be related with their regenerative ability, one could think that this is a general rule in which the hampering factor is a combination of proliferative capacity (i.e., retention of stem cell compartments) and tissue architectural complexity. Yet, studies carried out in the last few years reveal a far more complex situation, indicating how heterogeneous such an issue could be through the phylogenetic tree[22,43]. Limb regeneration in amphibians is a clear example of such a heterogeneity in closely related animal species; it occurs throughout life in newts and salamanders (urodele amphibians), whereas in frogs and toads (anuran amphibians), it is restricted to the developing larval limb[29]. As an example of heterogeneity in mammalian tissues, one can compare organs with very low physiological cell renewal, e.g., the liver, which perform remarkable regeneration after cell loss in the absence of clearly identified liver stem cell niches[51,52], with the central nervous system (CNS), which largely consists of nonrenewable tissue in spite of the existence of well-characterized stem cells and niches[53,54] (see next section for more detail). These examples show how indistinct the search could be for a hypothetical, shared logic that would account for regenerative processes, when considering stem cells through different animal phyla and in tissues showing different cell renewal rates and architectural complexity (Fig. 2).

Apart from the number and anatomical distribution of active stem cell niches in organisms, organs, and tissues (see next section), one possible explanation could reside in the different capacity of stem cells to regulate their active and quiescent states in complex organisms[55]. The latent regenerative capacity of stem cells in different tissues shows that the niche microenvironment determines whether or not they will participate in regeneration, form scar tissue, or remain dormant[56]. Unlike invertebrate stem cells that normally undergo constant cycling[57], mammalian adult stem cells are predominantly in a quiescent state[58,59]. It has been recently suggested that quiescent and active stem cell populations can coexist within tissues in separate, yet adjacent, locations where they cooperate in providing a mechanism to ensure high rates of physiological self-renewal as well as flexible damage repair[55]. According to these authors, the evolutionary advantage of such a coexistence could be linked to increasing animal life spans and body sizes, which in turn creates the need for increasing the longevity and output of adult stem cell pools in the rapidly regenerating tissues, without increasing the risk for mutations. Thus, in turn, an approach from a comparative point of view could improve our understanding of stem cell functioning in general.

All the examples reported above and taken from different representatives of the animal world introduce another step of variability in the regenerative capacity of complex metazoans: the striking differences that exist among different tissues. This refers to the question asked for animal species, now shifted at the tissue level: Why do some tissues regenerate, whereas others do not?

In the next section, such issues will be analyzed by taking into consideration different tissues in the mammalian body.

### Regeneration in Different Mammalian Tissues

In large and complex multicellular organisms as we are, an important issue concerning successful regeneration is the number and location of stem cell niches. Tissues and organs undergoing continuous cell renewal (e.g., skin, blood, cornea, many epithelia) do contain multiple and disperse units of stem cell niches (e.g., intestinal crypts, hair follicle bulge in the skin, endosteal and perivascular hematopoietic niches in bone marrow[60,61,62]; Fig. 2). After injury, regeneration in these labile tissues is favored by the persistence of undamaged stem cell niches. In addition, other sources of stem/progenitor cells have progressively been discovered, e.g., skin stem cells and progenitors in the interfollicular epidermis and in the sebaceous glands[62], or facultative hematopoietic niches at different locations in the bone marrow

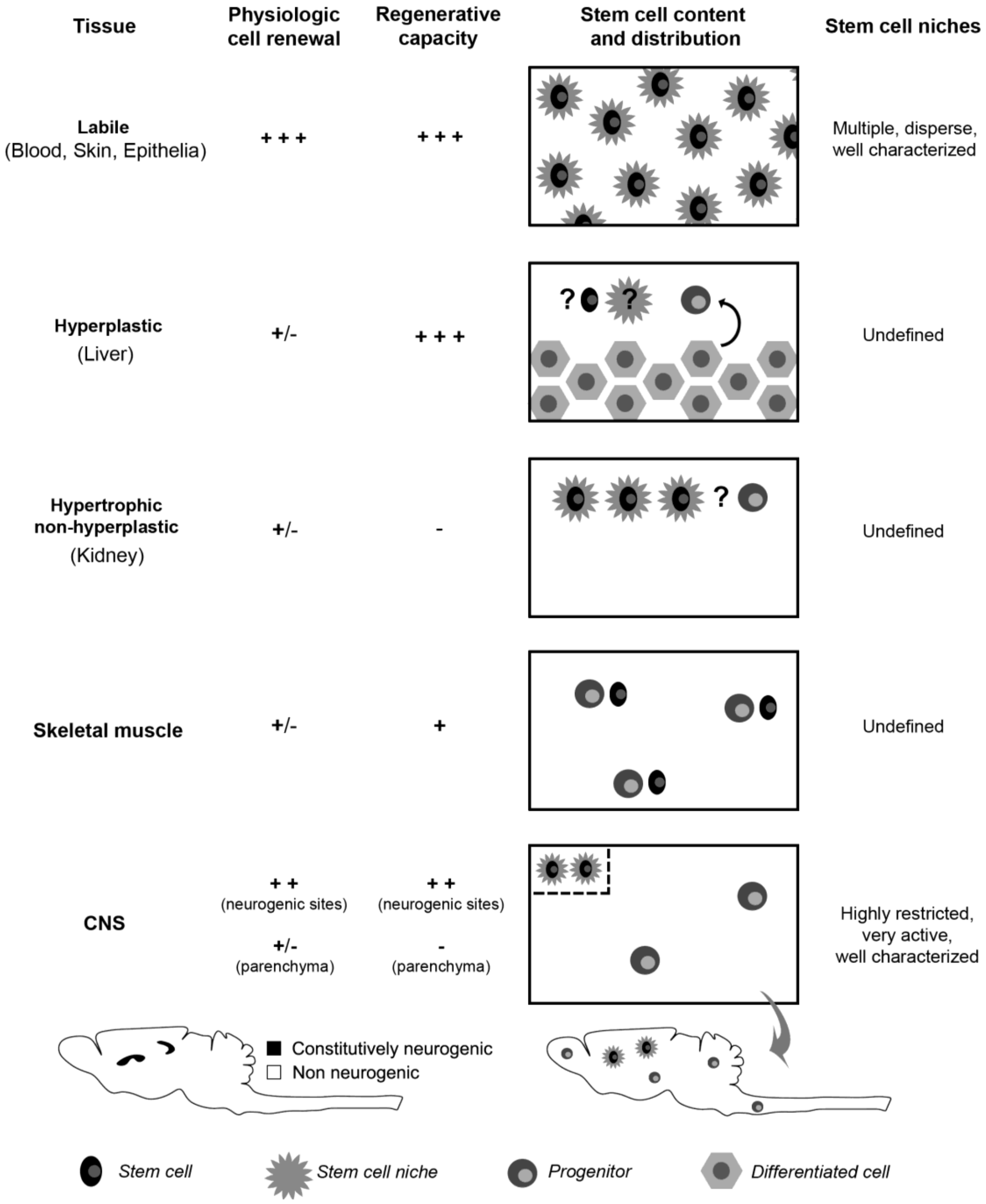

**FIGURE 2.** Different features of mammalian tissues endowed with different cell turnover and regenerative capacities, with particular reference to their content and distribution of stem/progenitor cells.

and in organs such as liver and spleen, which support the maintenance of similar niches. In metazoans, the ability to dynamically redistribute and activate new niches is an important strategy for regenerative capacity, which allows high potential for modulation in challenging physiological and pathological states[60,63]. By contrast, in some stable organs showing low cell renewal rates, but retaining remarkable potentialities for compensatory hyperplasia upon functional demands, e.g., kidney and liver, stem cells and their niches appear to be less active and have not been clearly characterized[51,64]. Although stemlike cells are thought to be present in the periportal regions of the liver[64,65], the so-called "liver regeneration" is carried out by proliferation of all the existing mature cellular populations composing the intact organ, then leading to restoration of the original lobule architecture[52]. In the kidney, podocytes can be replaced by a population of progenitors within the Bowman's capsule[66,67], yet no one cell can repopulate an entire kidney, as is the case for labile (e.g., blood) or other stable parenchymal tissues (e.g., mammary gland[68]). Also, the skeletal muscle is a largely postmitotic tissue endowed with low cell turnover; in the past, it was considered a perennial tissue along with the nervous system[49]. Now it has been shown that skeletal muscles maintain a remarkable capacity to regenerate after injury[43], a property granted by satellite cells resident beneath the basal lamina of myofibers that function as myogenic precursors during muscle regeneration[69,70]. Thus, skeletal muscles contain multiple and disperse stem/progenitor cells in the absence of well-identified, active stem cell niches[43,71] (Fig. 2).

Complexity of mammalian tissues/organs seems to go in parallel with high heterogeneity in the distribution/features of stem cell compartments. Accordingly, the CNS, which is endowed with true and very active stem cells that are topographically highly restricted within specific brain regions[53,72,73], is sharply different from both the context of multiple, distributed stem cell niches (labile tissues) and that of apparent absence of stem cell niches (hyperplastic and nonhyperplastic, stable tissues; see below and Fig. 2).

### *The Issue of Tissue Architectural Complexity is Not a Simple One*

The examples given above indicate that increasing complexity in animal structures does matter, but it is not enough to explain regenerative capacities. Basal proliferative activity and the existence of well-established, disperse stem cell niches are important premises for tissue regenerative ability, yet, alone, they cannot assure its occurrence in complex organisms. In other words, there is no linear and direct relationship between the regenerative capability of a tissue and its cell proliferation/stem cell activity.

An alternative approach to explain the heterogeneity of regenerative capacity among tissues considers its relationship with growth. Apart from some vertebrate species, e.g., some fish, that are characterized by indeterminate growth, mammals reach limited body sizes and some of their organs are not endowed with unlimited growth. This fact led R.J. Goss[74] to propose an alternative classification of tissues and organs with respect to that based simply on mitotic activity. Accordingly, tissues are viewed in terms of their functional units defined as "the smallest irreducible structure still capable of performing those specialized functions for which an organ may be adapted"[74]. The functional units can be represented either by the cell itself (e.g., blood cells) or by a histological entity made up of a number of cells that can perform their specialized physiological functions only as a team (e.g., follicles, nephrons, alveoli). The mammalian kidney is typically an organ incapable of true regeneration; although capable of hyperplastic responses after contralateral nephrectomy, this does not augment the number of nephrons[51] (Fig. 2). Tissues and organs of the body normally undergo the growth-regulating influences of functional demands, some being potentially capable of unlimited growth while others are not. According to Goss, this reflects a strategy in the way growth mechanisms have evolved, making the so-called "hypertrophic" organs incapable of hyperplasia. Some of the body's most vitally essential organs (heart, kidney, lung) lack the substantial ability to make additional structural units in the adult and are therefore hampered in regeneration[74,75]. The CNS, although not being a multitude of repetitive units, shares this functional property with hypertrophic, but not hyperplastic, organs (Fig. 2). In the second part of this review, we will see that the recent discoveries related to adult neurogenesis have only partially changed this view.

In summary, the environmental effects and regulatory factors regulating dynamic cell turnover in adult vertebrate organs are not yet thoroughly known. Understanding the intrinsic local and systemic factors that modulate the loops and feedback regulations of tissue growth and size is crucial both in regeneration and cancer research[76]. In the context of the present review, several factors are gathering that alone are not determinant for allowing/hampering regeneration, yet together they can contribute to opening either the regenerative or the tissue repair outcome.

### Regeneration and Repair

A fundamental concept in regenerative medicine resides in the fact that, after damage, in parallel with and/or in replacement of regeneration, repair can also occur. Indeed, complete (perfect) regeneration of complex tissues, and even of labile tissue in complex organisms, is usually precluded by fibrotic reactions that lead to scarring[3,19,21,77]. This opens a crucial question concerning the relationships existing between regeneration and repair, giving particular relevance to the understanding of factors that hamper regeneration in certain systems with respect to others, in parallel with the search for common genetic, cellular, and molecular mechanisms that do allow it. Studies on fin regeneration in zebrafish showing that a fish mutant devoid of blastema fails to regenerate the fin, but has normal wound healing responses[78], indicate that the two processes might differ at the cellular and molecular level[35]. On the other hand, some invertebrates (oloturia) and vertebrates (axolotl) employ analogous cellular mechanisms during wound healing and organ regeneration[35,79]. On these bases, Roy and Levesque[79] studied the links existing among three processes — regeneration, development, and wound healing — asking the question if some regeneration capacities are basically a process of superhealing. In their work, they reviewed: (1) the parallels between development and regeneration in axolotl (gene expression, pattern formation, nerve independency, cell differentiation), (2) those between such regeneration and the wound healing in adult mammals (inflammation, blood clot, wound epithelium formation, fibroblast migration, extracellular matrix restructuration, nerve dependency), and (3) aspects that are unique to regeneration (cell dedifferentiation, blastema formation). The results of this analysis underline the fact that similarities between regeneration and wound healing correspond to the first phase of regeneration (formation of wound epithelium and remodeling of the extracellular matrix), whereas those between regeneration and development reside in the second phase (redevelopment). Yet, cell dedifferentiation and blastema formation are peculiar to regeneration and are not observed in wound healing, thus suggesting that regeneration is not just an improved version of healing (see [79] for more detail).

Another emerging concept that could help to explain the regeneration/repair interplay is the negative effect that immune cells have on wound healing (reviewed in [77]). The immune system is of primary importance in determining the response of a tissue to injury, particularly the degree of inflammation and extent of scarring. This hypothesis is based on the fact that many components of the vertebrate immune system have evolved with dual, interrelated functions of both protecting injured tissues from infection and providing for tissue repair. Thus, the different capacity for organ regeneration through the phylogenetic tree would also be correlated with the specialization of the immune system[77,80,81]. The loss of regenerative capacity observed between urodele and anuran amphibians (the latter maintaining this ability only at the larval stage) is viewed as a turning point linked to the fact that urodeles are "immunodeficient" with respect to anurans[82]. The immune system of the anuran *Xenopus laevis* is ancestral at the larval stage, whereas it becomes similar to that of mammals in the adult[83]. In mammals, evolution of the immune system has produced inflammatory cellular interactions at sites of injury, thus optimizing tissue defense and repair, but leading to concomitant loss of regenerative capacity. In addition, studies on scar-free repair of fetal mammalian skin show how the potential to regenerate anatomically and functionally complete new structures is lost gradually during ontogeny, in coincidence with development of an immune system producing an inflammatory response in injured tissues[77,81].

In conclusion, increasing complexity in whole organism and tissue architecture could be an important factor for determining the maintenance or loss of regenerative/reparative processes in different animal phyla; yet, it is not enough. A further level of entanglement in the interplay among different variables regulating the issue of regeneration/repair can involve type and location of stem cells/niches, growth regulation, and immunotolerance response (Fig. 3). In the next section, this knowledge will be used to analyze the state of the art in the mammalian CNS; namely, one of the organs known to be most refractory to regeneration and healing.

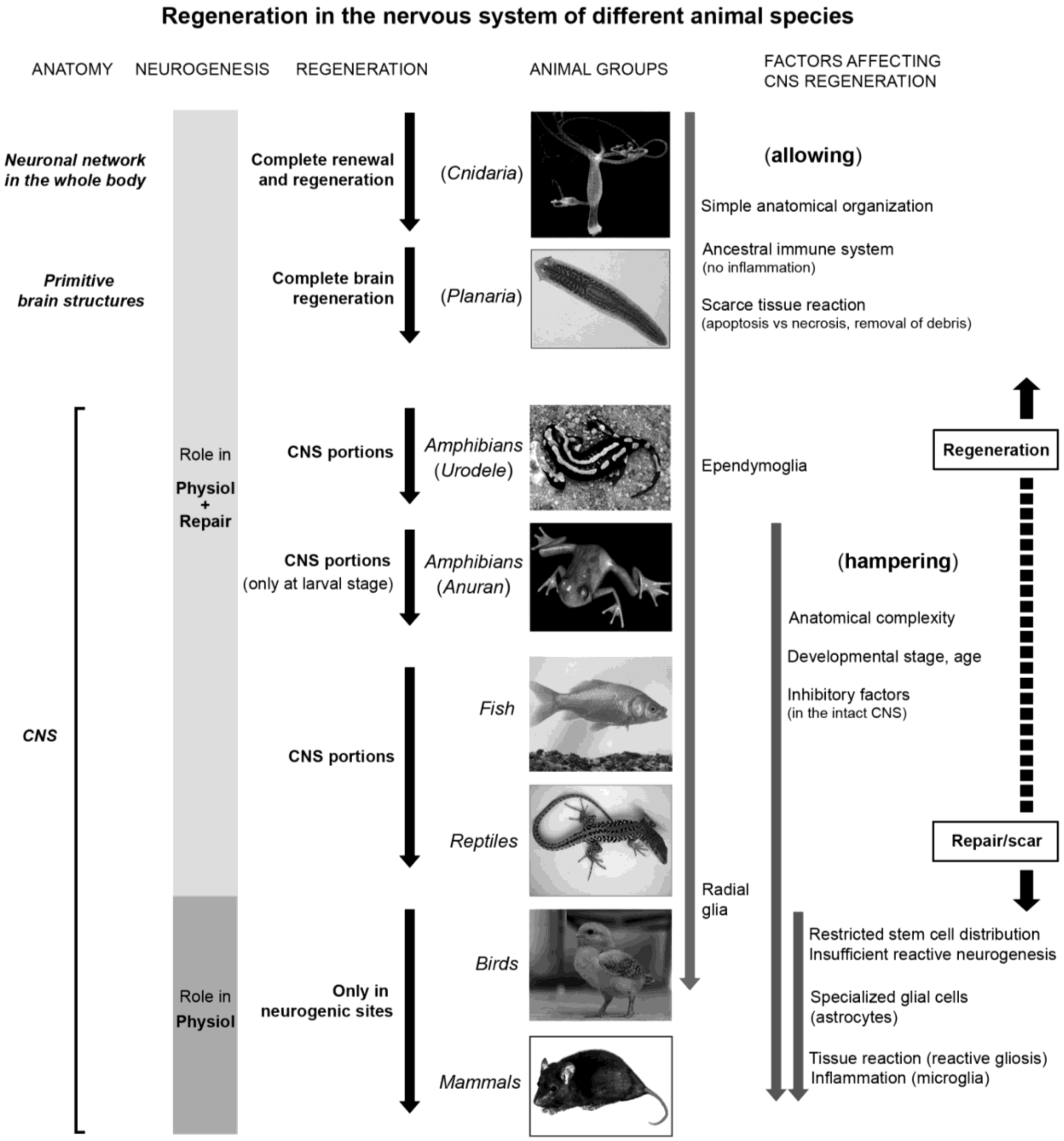

**FIGURE 3.** Schematic representation of the progressive restriction of regenerative capacity in the nervous system of different animal species, put in relation with several variables.

## THE FAILURE OF CNS REGENERATION IN MAMMALS

### Some Shadowy Concepts

#### *The Meanings of the Word "Regeneration" in the Nervous System*

With respect to other organs, the CNS shows structural peculiarities since neurons and glial cells possess ramified, intermingled processes, some of which project very far from the cell body. This fact makes the

CNS not simply a “mass” of organized cells, but a complex set of circuits in which a remarkable portion is composed of “cables” and synaptic contacts. For this reason, along with the old allocation of nerve cells as “perennial” (nonrenewable), the word «regeneration» in neuroscience was originally restricted to the regrowth/re-elongation of cell processes by surviving cell bodies after injury (e.g., axonal regeneration[84,85]). After the discovery of adult mammalian neurogenesis, the meaning of the word regeneration could be extended to that of new nerve and glial cell production[7,86]. Hidden in this phraseology are two important steps in the hypothetical “regenerative neurosciences”: (1) restoring lost connections from pre-existing neurons (axonogenesis and synaptogenesis) and (2) replacement of lost cells with newly generated elements coming from stem/progenitor cells (adult neurogenesis). Both these steps represent fundamental aspects of CNS structural plasticity and are complementary in the ultimate purpose of healing lost/damaged neural circuits[15,87,88]. Nevertheless, in the nervous system, plasticity and cell replacement can have different importance and functional meanings when regarded in an evolutionary perspective, as a consequence of many facts reported in the previous sections about general principles of regeneration.

### *The Need(s) for Nervous System Structural Plasticity*

The adult mammalian CNS can be considered as substantially static under the profile of cell genesis/renewal, although we know that it is remarkably plastic under the profile of neural connection reorganization, e.g., experience-dependent (structural) synaptic plasticity[89]. In fact, structural plasticity first allows the CNS to adapt its genetically determined anatomical organization (defined and assembled during developmental neurogenesis and then substantially unchanged as to the number and gross shape of cellular elements involved) to the challenging/changing environment in which the animal lives. Another type of plasticity, involving continuous genesis of neurons and glial cells, and ensuring physiological cell renewal and/or different degrees of regeneration, is common in the nervous tissue of invertebrates and many nonmammalian vertebrates. This higher level of structural remodeling fulfills a second potential need for plasticity in the nervous system: the capability of self-repair after damage. However, this problem is a harsh one when dealing with mammals since their CNS simply does *not* regenerate.

Twenty years ago, two important discoveries made us aware that (1) neural stem cells do exist in the adult mammalian brain[12,73] and (2) they account for adult neurogenesis throughout life within restricted brain regions[13,73], even in humans[90,91,92]. Such a possibility for cell renewal theoretically incorporates our nervous system into the issue of regeneration. Yet, even if the adult neurogenic zones can “regenerate” the whole stem cell niche after cytotoxic removal of proliferating elements[86], most of the mammalian CNS substantially remains a nonrenewable, perennial tissue. Determining how it is placed in the context of regenerative plasticity is a crucial point for developing new strategies to be pursued to figure out efficacious therapeutic approaches. In the particular case of humans, an improved health and wellness attained during recent history (the so-called “cultural evolution”) has created a new need for structural plasticity aimed at coping with the so-called “diseases of modernity”; namely, an increased incidence of neurodegenerative diseases and/or loss of neurons in parallel with prolonged life span[93].

In order to answer the many open questions, we should start with those concerning how CNS regenerative processes have evolved in different animals, taking into account the distribution of neural stem/progenitor cells (and their niches) granting regenerative/reparative potential in the nervous system of different species.

### A Lost Function?

As for other tissues of the body, the regenerative/repair capacities of the nervous system highly vary across the phylogenetic tree and there is now some substantial knowledge about how they have evolved (Fig. 3). In the oldest living metazoans, such as the cnidarian polyp hydra, the nervous system consists of a nerve net intermingled between the epithelial cells throughout the animal. Both cell types (epithelial and neural) are balanced in their production and loss, leading to an organization characterized by "general plasticity", which strongly supports regeneration[50]. Interestingly, neurons of the nerve net are continuously lost with the epithelial tissue, both cell types being displaced towards an extremity or into buds, and in parallel, new neurons arise constantly and are added to the net[94,95]. This organization is reminiscent of labile tissues in mammals in which many cells dying by apoptosis are continuously replenished with newly generated elements through the activity of multiple, disperse stem cell niches (e.g., intestinal crypts[96]). Primitive bilaterians, which start to show an asymmetric regionalization of the nerve net[97], and planarians, which possess a primitive brain structure (a sort of bilobed brain formed by two cephalic ganglia[98]), can still regenerate a functional brain from almost any tiny body fragment[25]. However, although some bilaterally symmetric animals, such as flatworms, annelids, and nemerteans, are renowned for their outstanding regeneration capacity, not all species of these taxa can regenerate equally well and some cannot regenerate at all[99].

Among vertebrates, some fish still exhibit a great potential for structural and functional regeneration of the adult brain and spinal cord after injury[31,32,33,34]. Imperfect regeneration can occur in the CNS of anuran amphibians and reptiles[100,101,102,103,104,105], although an almost complete recovery has been observed after certain types of lesion in the cerebral cortex of lizards[101,103] and in the midbrain of salamanders[104]. Similar to that described for limb regeneration, CNS regeneration differs in urodele and anuran amphibians, being possible during adulthood in the former, but restricted to the larval stage in the latter[81,102]. Molecular analyses showed that reaccess to embryonic developmental programs does occur in salamander and axolotl regenerative processes[106,107], whereas in mammals and birds, some domains become refractory to signals very early, which could provide another explanation for the loss of regenerative capacity[7]. The spinal cord regeneration in chicks, which occurs only at embryonic stages, is a clear example of transition between regeneration permissive and nonpermissive stages, the latter coinciding with the end of a "neurogenic stage" or "neurogenic environment"[108]; see below.

On the whole, by comparing regenerative abilities in different animal species, it seems obvious that this capacity is strikingly reduced going from animals with very simple nervous systems to mammals. Yet this statement is an oversimplification when taking into account the persistence of neurogenesis, at least for two reasons: (1) nerve cell renewal is present in all animals studied and (2) the olfactory bulb and hippocampus of mammals are highly complex structures. The real problem of comparative neurogenesis/regeneration is that such an approach is not an easy task since different species possess remarkably different nervous systems and neurogenesis has adapted differently to each environment, not always being strictly linked with regenerative capacities (see below). In addition, regeneration in the nervous system seems to be a by-product rather than a specific outcome of evolution.

On the basis of what is described in the previous sections, to understand how regenerative capacities have been lost in our CNS, we can try to put together three main, multifaceted aspects: (1) the peculiar complexity of the nervous system as a tissue and the different complexity of nervous systems in different animals; (2) the content, amount, and distribution of stem/progenitor cells and the (theoretically consequent) persistence of neurogenesis; and (3) other possible factors that can allow/hamper regeneration in the nervous system. Unfortunately, we can perform this analysis only when considering the few species that have been deeply studied as to their nervous system anatomy and plastic/regenerative capabilities among the millions of animal species existing worldwide.

### *Structural Complexity of the Nervous Tissue in Different Nervous Systems*

The reasons for considering anatomical complexity as a strong limiting factor to regeneration in the nervous system of complex metazoans are clear: in structurally simple animal models, a manageable number of neurons form simple circuits associated with specific functions. In the simplest types of nervous system organization (nerve nets connected by neurite bundles), only hundreds of neurons can be found[109,110]. Hence, after the CNS of a simple invertebrate organism has been cut in two, individual axons regrow to establish connections with appropriate synaptic targets[50,97,111]. By contrast, the huge number of cells existing in large mammalian brains (in humans estimated in $10^{11}$ neurons, with even more supporting glial cells and $10^{15}$ synapses[112], or $0.9 \times 10^{11}$ with an equal number of glial cells according to a more recent estimate[113]), along with complex neuronal circuits involving highly intermingled short, medium, long-distance axonal projections, play against successful regeneration.

Nevertheless, mammalian CNS complexity is not just architectural. Although composed of essentially three cell types (neurons, astrocytes, and oligodendrocytes), the nervous tissue is highly heterogeneous since it contains many subclasses of neuronal and glial cells that highly differ as to their location, morphology, and functions shaped by their individual developmental history[114,115,116]. As a consequence of such a “phenotypic” heterogeneity (*among* different CNS regions and *within* each CNS region), regenerative/reparative processes in complex CNSs can remarkably vary depending on the type and location of damage, which explains the extreme variety of outcomes encountered when studying regeneration in different animal species, CNS regions, and experimental paradigms.

The complexity of the CNS can also be increased with age in relation to body growth. Unlike insects that progress through larval and nymph or pupal stages before becoming adults that no longer grow, most of the larger species of crustaceans attain their adult form early in their development and then grow indeterminately, remarkably increasing in weight from juvenile through adult stages[117,118]. Unlike mammals, where muscle growth is based on an increase in size, but not in number, of muscle fibers, in some groups of fish, a continuous growth due to the addition of new muscle fibers into adult life is described[119]. This is one of the possible explanations for the continuous addition and replacement of neurons in the fish brain[120]. So, in several animal species, brain growth can be linked to persistent neurogenesis, which provides new neurons to cope with the increased size and surface area of the body, with particular reference to sensory and motor systems[103,120,121].

Finally, since it has been proposed that CNS regeneration described in vertebrates such as fish, reptiles, salamanders, and tadpoles occurs through reactivation of ventricular layers and consequent recapitulation of developmental morphogenic programs[7,103,105], it is not yet clear why such a possibility could have been lost in the CNS of other animals (e.g., mammals). On the other hand, by applying to the nervous system the notion that spontaneous regeneration is linked to whatever potentially embryonic remains within the adult body (substantially, in terms of stem cell niches), it is clear that the progressive restriction of the stem cell content, in terms of number, spatial localization, and favorable regulatory microenvironment, could be important in hampering regeneration in large CNSs endowed with low, restricted neurogenic activity.

### *Content, Amount, and Distribution of Stem/Progenitor Cells*

The trend of gradual decrease in the neurogenic abilities in neural systems is quite parallel to a topographical/numerical restriction of germinal layer–derived stem cell niches (Fig. 3). Neurogenesis sustained by multiple, disperse stem cell niches persists throughout the whole body of invertebrates[24,98]. Also, in some vertebrates such as fish and reptiles, adult neurogenesis can be found in wide CNS regions, including the cerebral cortex and cerebellum[32,101,121,122,123,124,125,126]. Yet, this is not a general rule since many exceptions are known. For instance, persistent neuroblasts of crickets are arranged in a cluster located at the apex of the mushroom body cortex[39], and precursor cells maintaining life-long proliferation in the brains of crayfish and lobsters reside within a specialized niche on the ventral surface of the brain[123]. Furthermore, whereas multiple stem cell niches and widespread germinal layers have been described in fish[32,121,122,123,124,125], the source of new neuroblasts seems to be confined in the ependymal layer of the ventricular “sulcis” in reptiles[101]. Also, in birds, neurogenesis starts from mitotically active periventricular layers, but newly generated cells can reach several telencephalic locations[127], whereas in mammals, the process is confined within two small neurogenic regions of the adult brain: the forebrain subventricular zone (SVZ) and the hippocampal dentate gyrus[53,72,73], and no newly generated neurons can reach the cortex (although some exceptions, not easily reproducible and still debated, have been described[128,129]). Persistent neurogenesis in mammals actually depends on stem cells residing within these zones as remnants of the embryonic germinal layers[53,130]. In the neural stem cell niches, under the control of a highly regulated microenvironment, they continue to act as proliferating cells that self-renew and generate a progeny of differentiating neuronal and glial precursors. As a consequence, the vast majority of the adult mammalian CNS, including brain, cerebellum, and spinal cord, should be considered as a nonrenewable tissue lacking regenerative capacity[6], wherein, unfortunately, most neurodegenerative diseases and ischemic/traumatic damage actually occur.

In spite of an apparent parallelism in the trends of neurogenesis and CNS regeneration in the phylogenetic tree (both progressively decreasing), many observations indicate that regenerative capacity is not always related in a direct way with endogenous neurogenic potential. In other words, although comparative studies indicate that a persistent neurogenic capacity is a phylogenetically highly conserved feature in the animal world[131], the rate of such a process, as well as its outcomes and functions, remarkably varies in different phyla and species. For instance, regenerative/reparative processes in the retina of different animals show some discrepancies; in amphibians, characterized by robust adult retinal neurogenesis and retinal regeneration, the neurogenic zone is not the major source of cells for regeneration. In addition, the mammalian retina is incapable of regeneration, yet some neurogenic processes are present[7, and references therein]. Also, regeneration in the zebrafish spinal cord is a striking phenomenon[31,132], yet spontaneous neurogenic processes are very limited in the intact spinal cord of these fish[133].

Hence, the persistence of neurogenesis could be a source for cell replacement, but it is not enough to ensure spontaneous regeneration in complex nervous systems, so other factors might be involved (Fig. 3). More specifically, the exact relationships existing between the “physiological” role of spontaneous neoneurogenesis and its potential benefit in brain repair/regeneration, the latter aspect disappearing when organisms of increasing complexity are concerned (see below; Fig. 3 and Table 1), are not yet clear. In other words, in mammals, the fact that damage to the adult brain can stimulate reparative neurogenesis does not imply that naturally occurring neurogenesis may be useful for brain repair[134]. More generally, such a fact is reminiscent of what is described above for regeneration in different tissues/organs: the apparent absence of a shared logic between the intrinsic proliferative/cell renewal capacity of a tissue (labile, stable, perennial) and its capability to undergo regeneration (Fig. 2). This again implies that additional factors might be responsible for allowing/restricting CNS regenerative processes.

### *Other Factors Allowing/Hampering Regeneration in the Nervous System*

On the bases of concepts illustrated above, it is easier to focus on the evolutionary success of CNS regeneration and on the mechanisms that allow it in some animals, whereas in mammals, the question could be shifted to the reasons for its progressive loss and on the causes of regenerative failure (Fig. 3).

As discussed in the paragraph on the relationships between regeneration and repair, in addition to anatomical/cellular aspects, a key role in supporting or inhibiting regeneration in the CNS has been attributed to the immune system[81]. Apparently, evolution led to a rather inefficient adaptive immune system in salamanders and a rapidly developing one in frogs, supporting the hypothesis that a strong immune surveillance coincides with loss of regenerative capacity[7]. A cross-link between the immune system and neural stem/progenitor cells to promote functional recovery is supported by accumulating evidence that the immune cells could also help to maintain adult CNS neurogenesis in physiological conditions[135,136,137,138,139,140]. Yet, this fact can be misleading since the response to tissue damage (largely linked to immunity) engages in reactive/reparative events that prevent regeneration in the CNS of mammals. By contrast, the high potential for structural and functional CNS regeneration exhibited by some nonmammalian vertebrates after injury is allowed by a sequence of well-orchestrated events that involve elimination of damaged cells by apoptosis and removal of cellular debris by the action

**TABLE 1**
**Variables involved in CNS Regeneration, Structural Plasticity, Repair, and Recovery**

| ANIMAL WORLD | | |
|---|---|---|
| **Factors negatively affecting CNS regeneration** | **Role of persistent neurogenesis** | **Role of stem cells** |
| Brain anatomical complexity and size<br><br>Scarcity of stem cells; restricted stem cell niches *(insufficient reactive neurogenesis)*<br><br>Specialized glial cells<br><br>Developmental stage<br><br>Aging<br><br>Inhibitory factors<br><br>Immune surveillance<br><br>Tissue reactions *(gliosis, cell death, inflammation)* | Adaptation to the environment *(olfaction, memory, etc.)*<br><br>**[All animals]**<br><br>Neural repair *(regeneration)*<br><br>**[Invertebrates, nonmammalian vertebrates]** | Renewal of spent cells in homeostasis<br><br>**[All animals]**<br><br>Cell replacement in response to injury<br><br>**[Invertebrates, nonmammalian vertebrates]** |

| MAMMALS | | |
|---|---|---|
| **Remaining structural plasticity in normal brain** | **Mechanisms of spontaneous recovery after damage** | **Possible actions for treating CNS damage** |
| **Cell replacement** | | |
| Adult neurogenesis *(neurogenic sites)*<br><br>Local, parenchymal progenitors *(potential neurogenesis)* | Regeneration *(limited to neurogenic sites)*<br><br>Reactive neurogenesis *(abortive)* | Replacing lost neurons and glia *(cell therapy, endogenous implementation) (rehabilitation?)* |
| **Modification of pre-existing cells/circuitries** | | |
| Axonal sprouting *(limited)*<br><br>Synaptic plasticity *(strength, formation, elimination)* | Restoration of function<br><br>Change in structure/function *(remaining undamaged neurons/glia)*<br><br>New behavioral strategies *(to accomplish the affected functions)*<br><br>Metabolic compensation | Removal of inhibition *(pharmacology)*<br><br>Actions at synapses *(rehabilitation)*<br><br>Enhancing intrinsic growth ability *(rehabilitation, pharmacology)* |

of microglia/macrophages, which precede the proliferation/migration/differentiation of endogenous neural precursors[35,141,142,143,144]. This fact has been shown for regeneration in lizard medial cortex[142,143] and in fish spinal cord[34,145,146]. Similarly, in reptiles, a scarce/absent inflammatory reaction present in tail regeneration contrasts with the massive inflammatory response in the limb leading to scarring[30]. Thus, another way to address the issue of "losing regenerative capacity" in complex metazoans should be that of taking into account the interplay among the endogenous neurogenic/regenerative potential and the activation of reparative events (discussed in the next paragraph).

When considering mammals, a further element should be added concerning the theory trying to explain the CNS failure in regeneration as a result of evolutionary constraints in which the injured CNS would not favor a strategy of regeneration, but rather one of minimizing further damage (e.g., by circumscribing the damaged area with a gliotic reaction[93]). In humans, such a hypothesis is also linked to the fact that the incidence of most neurodegenerative diseases is due to extended life span as a by-product of enriched life quality.

### The Reasons for Failure in Mammalian CNS Regeneration

The adult human CNS, in spite of the presence of stem/progenitor cells[147], is a highly complex structure wherein no regeneration occurs and glial scars form after injury, so that the reparative processes only leave a place for compensatory plasticity. A reasonable conclusion might be that the mammalian CNS regenerative capacity could have reached a point of nonreturn as a consequence of multiple, converging aspects: (1) a remarkable anatomical complexity, (2) the high topographical restriction of germinal layer–derived stem cell niches and consequent lower amounts of stem cells (insufficient reactive neurogenesis), (3) the emergence of specialized glial cell populations with respect to immature radial/ependymal cells, (4) the developmental stage, (5) the existence of inhibitory factors in the intact CNS, (6) a strong immune surveillance, and (7) the consequent tissue reactions, most of which are detrimental (e.g., reactive gliosis, cell death, inflammation) (Fig. 3 and Table 1).

Issues relative to points 1, 2, and 6 have been addressed in the above sections; the other points will be briefly discussed in the following paragraphs.

#### *Inhibitory Factors in the Intact CNS*

Several factors that are normally present in non-neurogenic regions of the intact CNS, especially in the white matter, mainly hamper axonal regeneration. Among them are myelin components, such as NogoA, myelin associated glycoprotein (MAG), oligodendrocyte-myelin glycoprotein (OMgp), and chondroitin sulfate proteoglycans (CSPGs, such as brevican and versican[141,144,148]). Others are negative axon guidance molecules (Sema 4D, Eph B3) and extracellular matrix proteins (CSPGs, such as neurocan, versican, phosphacan, brevican, Ng2, tenascins). Their effect may depend on the exposure of specific ligands/receptors on the cell surface. This fact could explain why axons of newly generated neurons can grow over long distances to their target[149,150] and axons of dissociated nerve cells injected in the adult rat white matter using a microtransplantation technique that minimizes scarring can rapidly extend for long distances[84,85]. In addition, plasticity in the adult CNS is restricted by perineuronal nets around neuronal cell bodies and dendrites, which appear at the closure of critical periods and contain several inhibitory CSPGs[151]. It is generally thought that both intrinsic and extrinsic factors cooperate in allowing/inhibiting cell/axonal regeneration in the highly heterogeneous CNS tissue[152,153].

Another aspect implicated in the inhibition of CNS regenerative potential (likely by modulating the factors listed above) is the developmental stage (postnatal and adult), since major changes that lead to incapability to regenerate in mammalian CNS appear after birth. Many cellular and molecular changes occurring in the postnatal period (e.g., cell differentiation and consequent molecular changes, extracellular matrix changes, etc.) converge in determining a still-undefined environment generally indicated as “tissue maturation”[111,154,155]. For instance, the sensitivity of neurons to MAG is developmentally regulated, with MAG-dependent inhibition beginning only in the early postnatal period[156]. Also, parenchymal astrocytes lose their stem cell properties after postnatal day 10 (P10) in mice[157]. The use of nonplacental species that are born in an immature state as animal models (e.g., opossum, corresponding to a 14-day mouse embryo at birth) support this view; in the spinal cord of opossums, fiber growth after lesions is allowed up to P9-12, then it stops abruptly[111], similar to what observed in birds[108]. A systematic and comprehensive analysis of factors involved in such a transition carried out in mammals at critical postnatal ages could be a promising research field for the future. The up- and down-regulation of gene expression during the brief developmental period when spinal cord regeneration stops, with and without experimentally induced lesions, has been recently analyzed in neonatal opossums[154]. Finally, another temporal factor generally acting at late stages is aging. It is well known that the brain neurogenic potential linked to stem cell niches can endure throughout life, yet with a substantial, progressive decrease of the rate of cell genesis[158,159].

### Tissue Reactions that Prevent Reparative/Regenerative Processes

In the damaged mammalian CNS, different types of reaction from non-neurogenic parenchyma take place instead of regeneration. When vascular, traumatic, inflammatory, or degenerative events occur, neurons and mature oligodendrocytes die or undergo regressive events, whereas astrocytes and Ng2+ cells (see below) set off an active response known as gliosis[160]. Reactive astrogliosis can exert both beneficial and detrimental effects in a context-dependent manner depending on different types of injury or disease[161]. More precisely, the gliotic process can be viewed as a gradated continuum of progressive cellular changes ranging from moderate forms, which exhibit the potential for resolution, to extreme levels of activation, which also involve cell proliferation and lead to scar formation[160,162]. The glial scar, predominantly consisting of reactive astrocytes and extracellular matrix molecules, is considered the major barrier for axon regeneration beyond the lesion site[85,163]. However, increasing evidence also suggests a beneficial role for scar tissue as part of the endogenous local immune regulation and repair process. First of all, the scar seals the injured site, thus avoiding the spread of neurotoxicity that causes secondary neuronal degeneration. Then it provides trophic support, scavenging, and vascularization, and acts positively against neurotoxicity. Furthermore, it has been suggested to regulate immune activity at the site of injury, by regulating the number of infiltrating cells and their spatial localization[161]. On these bases, Rolls et al. propose that a scar has beneficial effects at certain phases of the recovery process, i.e., the acute phase (hours to days after injury), and destructive effects at others, such as the subacute and chronic phases (weeks and years). Hence, the frequent assertions that outgrowth of axons in the damaged CNS is prevented by inhibitory molecules and that the glial scar is responsible seem unlikely to be the complete explanation, since scars develop late, long after fibers have had the opportunity, but failed, to grow across the lesion[111]. All these aspects are topics of an open field of research, since understanding the mechanisms and timing of the aforementioned processes could have remarkable therapeutic implications.

Among events occurring in the reaction from non-neurogenic tissue, cell death also plays a role. Unlike nonmammalian vertebrates, necrosis is the predominant type of cell death in mammals, thus leading to inflammation and triggering further necrotic events at the site of the injury[164,165]. Much of the permanent damage that occurs in response to mammalian nervous system injury, in addition to direct tissue reaction, is mediated by endogenous secondary processes (excitotoxicity, oxidative damage, and inflammation). Thus, unlike nonmammalian vertebrates (see the example of fish in the above sections), in mammals, the healing process produces signals largely restrictive or nonsupportive for regenerative events. Such responses are thought to be the result of unavoidable evolutionary constraints (for this interesting theory, see [93]), so that in complex metazoans, the role of immunity in the CNS is unclear: it is required to eliminate dangerous/degenerated material and to support regeneration, but also causes significant parenchymal damage. Since early transplantation experiments demonstrated remarkable tolerance to various grafts performed in the CNS, this “immunologically privileged status”[166] was interpreted as reflecting evolutionary pressure to down-modulate certain actions of immune cells within particularly vulnerable tissues. Yet, following a new emerging view, in the mammalian CNS, the immune-mediated reactions can exert both beneficial and detrimental effects in a context-dependent manner depending on the types of injury, the time following injury, or the different disease conditions[167]. In conclusion, it is now generally recognized that the CNS requires both innate and adaptive immune responses for its maintenance, repair, and renewal; nevertheless, in mammals, these responses must be tightly controlled.

### The Role of Glia

Research work carried out in the last decade has revealed that radial glial cells are key players in neurogenesis[168]. In mammals, these cells deeply transform postnatally, giving rise to specialized ependymal and astrocytic cells[53,169,170]. Importantly, they have been recognized as the embryonic counterpart of adult neural stem cells following transformation into SVZ astrocytes[170]. In nonmammalian vertebrates, glial cells are less specialized and radial glia can persist during adulthood as an intermediate, highly plastic cell type called ependymo-radial glia (or radial ependymoglia) that are involved in regeneration[143,171,172]. For instance, spinal cord regeneration that occurs in the injured adult zebrafish, even including motorneurons, starts from ventricular zone proliferation of olig2+ ependymo-radial glia progenitors[132]. Similarly, the lesioned midbrain of adult salamanders can regenerate by reactivating proliferation of radial glia-like ependymoglia cells[100,104,173], and radial glia-ependymocytes of the lizard medial cortex participate actively in the removal/phagocytosis of cellular debris generated in the lesion[143].

The overall organization of astroglial structures is somehow linked to the macroscopic structure of the brain[174], although a clear logic in its evolutionary history is still lacking. In anamniote vertebrate species, such as fish, either radial ependymoglia (tanycytes) or astrocytes might be predominant. Among amniotes, astrocytes prevail in some species, a fact that can be seen by comparing the “tanycytic” brains of reptiles to the “astrocytic” brains of birds and mammals[175,176,177,178,179]. Replacement of radial ependymoglia with astrocytes in different phyla is interpreted as linked either to the thickening of the brain wall (with increased surface/volume ratio of the cells that must be replaced by astrocytes) or to an increasing complexity of the vascular network that requires the appearance of astrocytes (references in [174]). The emergence in mammals of specialized glial cell populations, such as astrocytes and ependymal cells, with respect to immature radial/ependymal cells, can be another factor hampering regeneration. Although playing no roles in spontaneous adult neurogenesis, mammalian ependymal cells are activated after injury[180], thus introducing the concept that even CNS cells not directly involved in the homeostatic neurogenic process can be “activated” in altered states (and thus taking part in the so-called “reactive neurogenesis”). The study of the multifaceted roles of astrocytic glial cells as stem/progenitor cell elements in brain homeostasis and reactive gliosis is another promising research field in mammalian CNS regeneration[162].

### What Remains?

The first part of this review analyzed how regeneration capability can be reduced, taking into account animal species, organs, tissues, and stem cells, then specifically focusing on the striking reduction observed within the nervous system. In the second part, at the extreme of a trip through the narrowing roads of regeneration along the phylogenetic tree, the reasons for regenerative failure in the mammalian CNS were discussed. Now, attention should be focused on which aspects of structural plasticity actually remain in our brain.

It is important to consider that cell replacement and regeneration can occur in two contexts: (1) renewal of spent cells during tissue homeostasis and (2) in response to external injury. Whereas these two aspects are considerably overlapping in nonmammalian vertebrates, adult neurogenesis in mammals seems to be restricted at the former (Fig. 3). For this reason, we should look at the mammalian brain neurogenic processes by separately considering their physiologic (homeostatic) role from their potential implication in brain repair. This approach could allow us to examine “realistically” such potential in mammals, by knowing the limits. Accordingly, I think that the more the brain neurogenic/regenerative potentialities become understandable in their homeostatic functions, the more new, trustworthy research lines could be addressed in the perspective of developing new therapeutic strategies for brain repair.

#### *Reactive Neurogenesis and Gliogenesis*

The possible activation of an endogenous neurogenic potential after a lesion in the mammalian CNS is a highly promising, yet controversial, issue. With “reactive neurogenesis”, the lesion-induced, *de novo* production of nerve and glial progenitor cells in the nervous tissue is intended. Such a reaction can occur both from neurogenic (stem cell–containing, germinal layer–derived areas) and non-neurogenic (progenitor cell–containing parenchyma) regions[172]. Nevertheless, the study of reactive neurogenesis should be addressed after having unraveled the real potential of the normal CNS structural plasticity as to its mechanisms and neurobiological significance, not yet utterly attained. Hence, the state of the art concerning neurogenic potential in the entire mammalian CNS will be briefly reviewed.

A "constitutive" neurogenesis is intended as a spontaneous ("actual") genesis of neurons and glia, and occurs primarily in small germinal layer–derived neurogenic sites endowed with stem cell niches[5,6]. In addition, in the remaining CNS parenchyma, local progenitor cells do support a "potential" neurogenesis, which does not manifest fully and spontaneously *in vivo*[5]. Progenitor cells that retain some proliferative capacity have been identified in most of the mature CNS parenchyma[171,181,182,183], although the origin of the "local" progenitor cells involved, as well as their fate, is still a matter of debate[184]. In the adult mouse, the largest class of these cells expresses the nerve/glial antigen 2 proteoglycan (Ng2)[171,185]. They are also called *polydendrocytes*[182] or *synantocytes*[181,186] and are morphologically, antigenically, and functionally distinct from mature astrocytes, oligodendrocytes, and microglia. Yet, in spite of their proliferative capacity and retention of potentialities *in vitro*, the Ng2+ cells do not perform neurogenesis *in vivo*, their commitment being restricted to the oligodendrocyte lineage during postnatal life and after neurodegeneration[187,188,189,190]. Unexpectedly, in some CNS regions of other mammalian species, local parenchymal progenitors actually sustain spontaneous neurogenesis *in vivo*[6,191,192]. In mice, some newly generated neocortical neurons have been found[128], as well as some neurons of the piriform cortex originating from Ng2+ progenitor cells[193]. The production of new neurons in the adult rabbit striatum[191] and cerebellum[192] suggests that regional neurogenic potential can vary among mammals.

After defining the neurogenic potential of the adult mammalian CNS, an important question is: How do the neural stem/progenitor cells behave in different injury/pathological contexts? Although topographically restricted, mammalian brain neurogenic sites that are active throughout life can react to injury. For instance, experiments carried out in rodent models of stroke (transient global ischemia and middle cerebral artery occlusion) revealed that reactive neurogenesis does occur from the SVZ, leading to increased cell production and migration of neuronal precursors to the lesion site[194,195]. In rats, it has been shown that the SVZ can redirect newborn neurons fated to the olfactory bulb to the injured areas within the striatum, yet generating only a few medium spiny neurons[194]. Induction of neurogenesis has been reported in the striatum, hippocampus, and corticospinal system[150,194,195,196,197,198]. Adult neurogenesis may also be substantially augmented in neurodegenerative diseases[199,200]. Nevertheless, although replacement of a few lost neuronal cells has been reported[149,150,194], the great majority of the newborn cells survive <1 month, and fail to replace lost neuronal populations and to restore damaged neuronal circuits[201]. In animal models of experimental autoimmune encephalomyelitis, the mobilization from the SVZ of neural precursors capable of differentiation into oligodendrocytes in the corpus callosum has also been reported[202,203].

In addition to reactive neurogenesis from stem cell–containing neurogenic zones, accumulating evidence indicates that different paradigms of brain lesion can induce neurogenic events from the local progenitors resident in normally non-neurogenic sites, including the neocortex and the hippocampus[172]. In one case, this was observed after selective neuronal death, thus supporting the hypothesis that potential regenerative processes of the adult brain can have more chances to succeed in the absence of tissue reaction[149]. This situation could be in line with the observation that in nonmammalian vertebrates capable of some CNS regeneration, damaged cells and debris are easily eliminated by apoptosis and by microglia/macrophages, thus leading to less tissue reaction[34,120,142,146] (see above). In another case, local progenitors that are in a relatively quiescent state in layer I of the rat cerebral cortex were activated after ischemia, giving rise to new cortical interneurons[204]. These examples support the hypothesis that the mature CNS parenchyma may retain a latent stem/progenitor cell potential, normally inhibited *in vivo* that, if properly evoked, might be exploited *in situ* for cell replacement[167,188]. Among local, parenchymal progenitors, some are oligodendrocyte progenitor cells (OPCs) that can generate oligodendrocytes in the mature CNS, thus serving as the primary source of remyelinating cells in demyelinated lesions[187]. In contrast to the situation that generally follows loss of neurons, CNS remyelination might be considered an effective regenerative process[187,205]. However, the term *myelin repair* evokes thoughts of a damaged, but otherwise intact, myelin internode being "patched up", a process for which there is no evidence. It thus does not emphasize the truly regenerative nature of *remyelination*, in which there is restoration of the prelesion cytoarchitecture[205]. Some capability for remyelination has been preserved in the human CNS and this is important in therapeutic perspectives since myelin repair prevents axonal degeneration[206]. If this occurs through stem/progenitor cells giving rise to new oligodendrocytes (either by pharmacologically induced recruitment of oligodendrocyte precursor cells or by intravenous injection of neural stem cells), it could be considered as a form of CNS regeneration[206,207]. Nevertheless, since newly made myelin sheaths are thinner than normal and have shorter internodes[208], it should be more appropriate to consider it a form of tissue repair. In addition to cell replacement (that at present remains quite theoretical), transplanted neural stem and progenitor cells can play a beneficial role in animal models of myelin disorders, especially through a bystander effect[167]. Yet, from a review dealing with evolutionary and comparative aspects of regeneration, we should keep in mind that many differences that exist between rodents and humans (e.g., difficulties to commit human neural stem cells towards the oligodendroglial lineage, incomplete knowledge of human neurogenic potential) still hamper human cell-based therapy for these diseases[209].

On the whole, it can be concluded that the brain has an endogenous regenerative potential and that in some pathological conditions, it becomes more permissive to neurogenesis/gliogenesis. Yet, in most cases, such potential cannot manifest in a successful way since the mobilization of neural progenitors is insufficient and inefficient to grant CNS regeneration/healing in most types of injury and/or disease, especially in relation with the inhibitory and nonpermissive aspects discussed above. Putting together the regenerative potentialities and the inhibitory factors playing against them, attention should be put on CNS regional differences *in vivo*, underlying the importance of the microenvironment for normal and lesion-induced neurogenesis, rather than on the regionally different properties of precursor cells[172]. Such heterogeneity is further increased by the multifaceted responses depending on the nature of CNS injuries: localized (e.g., trauma, ischemia) or multifocal (e.g., Alzheimer's disease, multiple sclerosis), acute (e.g., trauma, ischemia), and progressive (most neurodegenerative diseases). Thus, CNS potentials for regeneration should be analyzed (and tackled) by taking into account their heterogeneity.

### Forms of Plasticity Alternative to Regeneration

As a consequence of the anatomical and evolutionary constraints discussed above, in the absence of regeneration, most plasticity enduring in the adult mammalian CNS might be confined within the existing connectivity. Specificity of connectivity, determined by intrinsic programs of gene expression that define cell phenotypes and by activity-dependent mechanisms, is essential to nervous system function. Nevertheless, many experiments reveal that cortical axons maintain the capacity to grow and branch in the normal adult neocortex[89]. Their relevance to experience-dependent plasticity remains unclear, although some indications have been obtained in the hippocampus that axonal sprouting occurs during spatial learning[210] or in response to environmental enrichment[211]. Most substantial forms of compensatory structural plasticity after a lesion consist of modified synaptic strength, synaptic formation/elimination, and axonal sprouting/pruning, all involving pre-existing cells and circuits. Of course, similar to what is seen for reactive neurogenesis and due to the extreme heterogeneity of CNS neural tissue and circuitries, the outcomes of lesion-induced compensatory plasticity would be highly variable, exiting into a wide range of events depending on the specific site and type of injury[212,213,214,215,216,217]. A clear picture is still lacking with regard to what exactly happens in the fine structure of most regions of the nervous tissue, a task requiring the combination of time-lapse imaging with ultrastructural analysis. Long-term *in vivo* imaging studies provide a complex picture of structural plasticity in some regions of the adult mammalian brain as a consequence of both experience and injury. On the whole, the large-scale organization of axons and dendrites is remarkably stable for most of the animal life span; nevertheless, a subset of synaptic structures can display cell type–specific, experience-dependent structural plasticity; the dynamics of this turnover being regulated by sensory experience[89]. The same approaches are beginning to reveal the mechanisms underlying spine abnormalities related to specific brain disorders. For example, in mice, dendrites become plastic in the vicinity of a cerebral infarct and these structural changes might provide a substrate for the long-term functional changes in the representational cortical maps that are observed after stroke[218].

A further level of plasticity consists of structural changes that involve the whole cell shape (both in neurons and glia), in some cases extended to entire cell populations or areas of the parenchyma endowed with high amounts of molecules usually abundant during embryonic development, but still expressed at specific sites in the adult CNS (e.g., the polysialylated form of the Neural Cell Adhesion Molecule, PSA-NCAM[219]). Such a feature is part of the normal, homeostatic and adaptive structural plasticity, e.g., activity-dependent synapse remodeling and neuroglial plasticity[220,221], but can be modulated after injury[222]. In addition, it has been shown recently that some prenatally generated neurons in the adult cerebral cortex maintain an immature state during adulthood by expressing PSA-NCAM and displaying a few synapses on their membrane[223], while others are mature neurons that not receive synapses at all[224], both possibly representing a reservoir of cells to be recruited at different postnatal ages for establishing new connections. In spite of remarkable advancements in unraveling mechanisms and consequences of such plastic changes, we still lack a detailed and systematic mapping concerning brain structural plasticity responses that are alternative to regeneration in the mammalian CNS affected by different injury/pathological contexts. It is worth mentioning that some of these “alternative” forms of plasticity, similar to those involving regenerative potential, are developmentally controlled, yet with critical periods that extend far beyond the early postnatal period (up to 35 days in rats and 5 years in humans; see, for example, [225]).

Coming back to the dichotomy existing in mammals between physiologically relevant capacities for structural plasticity and lesion-induced reactive/reparative events, a possible explanation has been proposed by Nicholls and collaborators[111]. These authors, starting from the question — What biological advantages could mammals have from not being able to repair the CNS after injury? — answer with an interpretation that is opposite to the common thought that the failure is the price to pay for having a highly complex CNS, by raising the possibility that failure is the penalty paid for having a highly plastic nervous system. They quote the example of frogs, which can regenerate their optic nerve and can see again, yet never learn to correct their behavior. For example, if the eye that has been removed is inserted upside down, the animal always jumps for a fly in the wrong direction[226]. In mammals, plasticity through axonal sprouting is permitted to mediate adaptation to novel stimuli, but only over short distances, so that one could speculate that such growth has to be limited in order to prevent chaotic connections from being formed in the brain. Thus, the issue of mammalian CNS complexity as a mix of the seven points playing against regeneration (see above) would be only partially compensated by structural plasticity mostly confined within the existing connectivity. Knowing what remains in terms of such plasticity (and, in parallel, trying to unravel how this situation has been reached in mammals during evolution) should be a mandatory step to correctly address the issue of brain repair and its therapeutic approach. Unfortunately, the two worlds of “regenerative neuroscience” and “rehabilitative neurology” still remain somehow disconnected.

### *Looking for a Substrate for Developing Therapeutic Perspectives*

By considering the limits and potentialities of our brain, two main therapeutic strategies can be figured out in CNS repair: (1) the exogenous delivery/endogenous implementation of new cellular elements (cell therapy) and (2) the rescue/plastic adaptation of pre-existing cells and circuits (obtained through rehabilitation). Both approaches might be modulated/implemented through pharmacological treatments[213,227].

As to the former approach, in parallel with an intense effort to produce stem/progenitor cells that could be used as transplantation tools in order to replace tissue elements that are lost/damaged in pathologies[14,15,228], a new perspective consists of getting insight into the potentialities of resident, adult stem/progenitor cells[6,149,150,162,172,187,188,204]. In both these directions, new attention has to be placed on the "context" (physiological and pathological) in which the cells live and work, starting from the theory that stem cells are functional states rather than physical entities, or at least from the fact that their behavior and differentiative fate is strongly influenced by the local environment[229]. When speaking about stem cells, such a microenvironment is the stem cell niche. The niche preserves cell balance within a basic unit of tissue, saving stem cells from depletion and from abnormal proliferation, and also modulates their response to physiological and pathological challenges[60,229,230]. Thus, the stem cell niches as "entities of action"[229] could be a key element in the realization of regenerative medicine, yet their occurrence and locations remarkably vary in animal tissues and are highly restricted in the mammalian CNS. Hence, in parallel with a deeper knowledge of the cell biology of different CNS progenitor cells, an open field of research for the future could be the search for "atypical" niches, in terms of the parenchymal environments possibly allowing some regenerative/repair processes in pathological states. An apparently unattainable problem is the fact that CNS regenerative potential (restricted in neurogenic sites and quiescent in the parenchyma), once activated, appears mostly uncoordinated and abortive, in some cases producing detrimental effects on regeneration/repair. This resumes, in turn, the concept of "tissue contexts" as fundamental players in regulating the progenitor cell behavior.

Particularly in the nervous system, the cells and the context in which they live can be strikingly modulated by the external environment in which the animals live[89,134,231]. For this reason, while expecting new, innovative stem cell–based therapies that could sustain a *de novo* structural plasticity, the current best approach is to lessen the physical/cognitive impairments and disabilities of patients with stroke, multiple sclerosis, spinal cord or brain injury, and other neurologic diseases through rehabilitation[232,233]. In the absence of regeneration, recovery in the CNS could occur by multiple mechanisms: (1) restoration of function of damaged neurons, (2) change in structure and function of remaining undamaged neurons and glia, (3) formation of new behavioral strategies to accomplish the affected functions, and (4) metabolic compensation, e.g., remaining substantia nigra neurons that increase their turnover of dopamine in a rat models of Parkinson's disease[134,234]. Sensory and motor functions that are mapped somatotopically have been demonstrated to change, other than in response to experience, particularly in development, also in adults after injury. For instance, recent imaging experiments tracked dendritic spines in the motor cortex during motor learning. Training in a forelimb reaching a task rapidly induces the growth of new dendritic spines, which are preferentially stabilized by subsequent training sessions[235]. Such changes occur in both animal models and human clinical trials, yet one must be cautious when comparing results obtained with invasive and noninvasive techniques[234]. Rehabilitative approaches should drive nervous system plasticity in a desired direction, i.e., the enhancement of function-enabling plasticity and the prevention of function-disabling plasticity. In order to reach this goal, rehabilitation can be associated with several complementary modalities, i.e., pharmacological interventions, environmental enrichment, and stem cell transplantation, to realize the so-called "restorative neurology"[236]. This could be imagined as a sort of functional brain repair occurring in the absence of true (perfect) regeneration, yet involving and enhancing/optimizing all structural changes that are allowed in the mammalian CNS. Some examples of environmental stimulation positively affecting the recovery by an additional effect on reactive neurogenesis are emerging. For instance, increased production of neuronal precursors has been observed both in the SVZ[237] and in the hippocampus[238] of postischemic rats maintained in an enriched environment or undergoing sessions of skilled reaching training, respectively.

In this perspective, a distinction may be made between factors that are not amenable for manipulation (e.g., age, type of injury, genetic factors) and those that are modifiable variables (environment, pharmacological agents, stem/progenitor cell transplantation and implementation), the latter being of interest for restorative neurology. Many papers deal with candidate drugs that specifically target adult neurogenesis and neural stem cells (so-called neurogenic drugs), sometimes referred to as "highly potent and highly specific for treating neurological diseases and disorders”. This seems quite an overestimation of the actual possibility for the modulation of brain repair, especially if taking into account the many constraints highlighted in this review. Good results have been obtained with molecules that interfere with inhibitory factors. For instance, blocking NogoA with antibodies (or interfering with the receptor), as well as administration of inosine (a factor responsible for the regeneration of fish retinal axons), can lead to enhanced sprouting[151]. Plasticity can also be reactivated in the adult CNS by treatment with chondroitinase ABC, which removes glycosaminoglycan chains from CSPGs[227]. However, at present, it is not clear which aspects of plasticity are enhanced by chondroitinase, anti-NogoA, and inosine, nor is it known in detail which connections are formed and changed. Particularly, promoting plasticity by itself does not necessarily bring back useful behavior, unless connections are stabilized and inappropriate connections removed. The approaches that combine several aspects could be the most promising. For instance, by combining a daily rehabilitation treatment (specific for the behavior that is to be enhanced) with pharmacological treatment (e.g., chondroitinase), a much greater recovery than either treatment alone is produced[227].

Thus, the next questions could be: Do rehabilitation and enriched environment affect the CNS regenerative potential in mammals other than affecting compensatory structural plasticity? And consequently: Is this effect restricted to the stem cell–containing neurogenic zones or can it be more widespread in the nonconstitutively neurogenic parenchyma? Many studies indicate that keeping physically active may be vital to brain health, since exercise promotes neurogenesis, increases brain volume, and improves cognitive function, and generally can help the aging brain to retain plasticity[231]. What appears interesting in the perspective of rehabilitation, especially in old age–related neurodegenerative diseases, is the link among physical exercise, brain structural plasticity, and cognition. The precise mechanisms by which exercise accomplishes these changes are not clearly understood. The involvement of the immune system has been hypothesized to explain why our body can influence our mind[140], yet this is really a very wide open field of research.

In conclusion, new lessons learned from basic research in neurogenesis/neural repair, including the comparative analysis of regeneration and persistent neurogenesis in different animal phyla, and the study of stem cell niches and parenchymal progenitors in the mammalian CNS, although not providing immediate therapeutic approaches, indicate some important facts that should be taken into account. First is the dichotomy that exists between the homeostatic role(s) of naturally occurring neurogenesis and the need for brain repair. Then are the other aspects that deserve deeper investigation: the complex role of the immune system, the nebulous overlapping between gliogenesis and neurogenesis, and the relationship between cell-specific and environmental factors affecting the reparative processes in each region of the CNS. Finally, a real challenge for the future could be that of finding an integration between the two main aspects of brain healing in mammals: the compensatory adaptation of pre-existing circuitries and the addition of new neuronal/glial cellular elements.

### ACKNOWLEDGMENTS

This work was supported by Compagnia di San Paolo (Progetto NEUROTRANSPLANT) and Regione Piemonte. I wish to thank Paolo Peretto and Daniela Carulli for critically reading the manuscript.
